# Supplementary material for: Strain variation in Bacillus cereus biofilms and their susceptibility to extracellular matrix-degrading enzymes
Source: PLoS One. 2021 Jun 16;16(6):e0245708. doi: 10.1371/journal.pone.0245708 (PMC8208538; doi:10.1371/journal.pone.0245708)

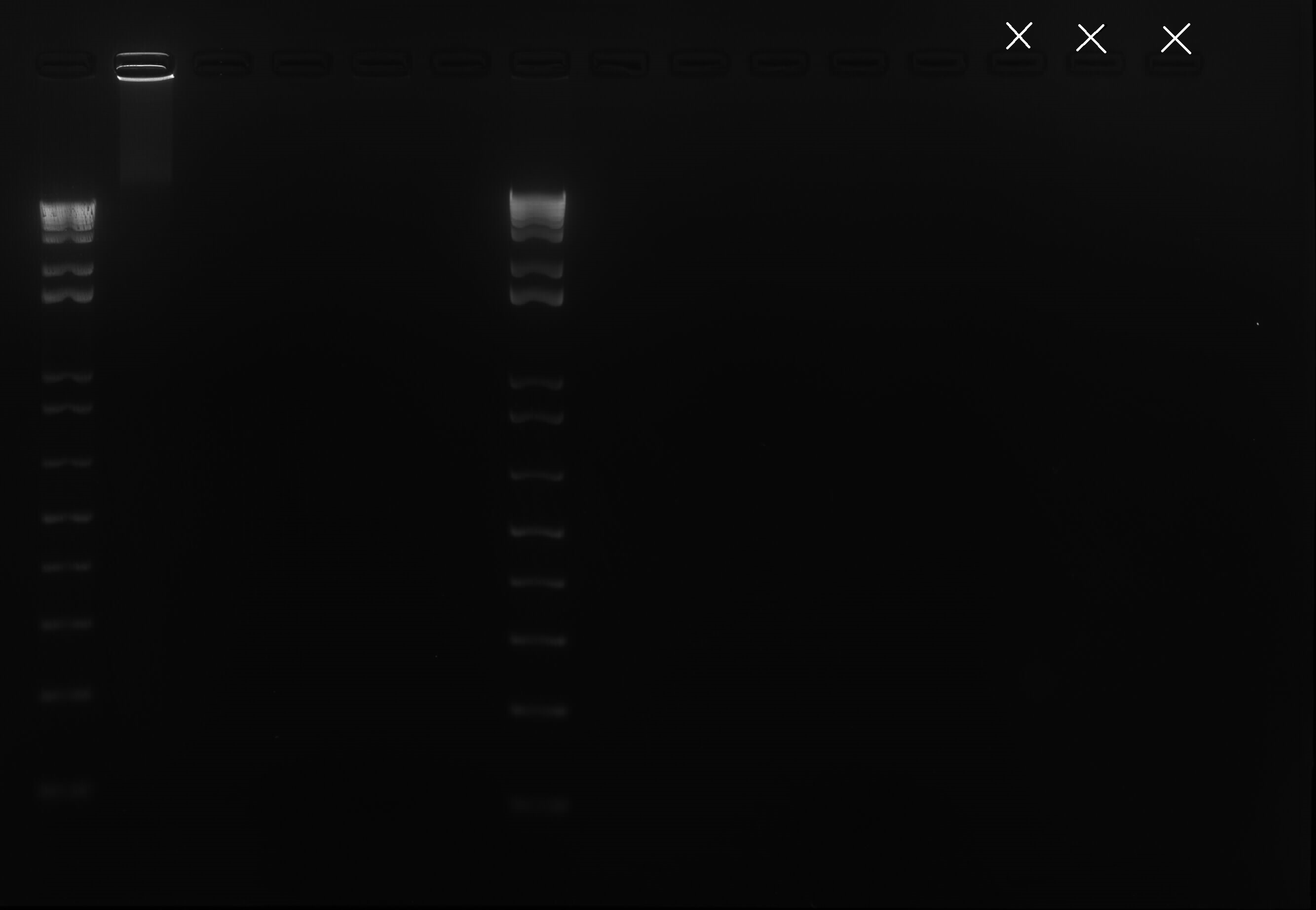

Supplement: S1 Fig — (PDF) [file pone.0245708.s001.pdf]
