# Supplementary material for: Strain variation in Bacillus cereus biofilms and their susceptibility to extracellular matrix-degrading enzymes
Source: PLoS One. 2021 Jun 16;16(6):e0245708. doi: 10.1371/journal.pone.0245708 (PMC8208538; doi:10.1371/journal.pone.0245708)
Supplement: Attachment — Submitted filename: Response to Reviewers.docx [file pone.0245708.s002.docx]

PONE-D-21-00279
Strain variation in Bacillus cereus biofilms and their susceptibility to extracellular matrix-degrading enzymes
PLOS ONE

Dear Dr. Kim,

Thank you for submitting your manuscript to PLOS ONE. After careful consideration, we feel that it has merit but does not fully meet PLOS ONE’s publication criteria as it currently stands. Therefore, we invite you to submit a revised version of the manuscript that addresses the points raised during the review process.


Please submit your revised manuscript by Mar 27 2021 11:59PM. If you will need more time than this to complete your revisions, please reply to this message or contact the journal office at plosone@plos.org. When you're ready to submit your revision, log on to https://www.editorialmanager.com/pone/ and select the 'Submissions Needing Revision' folder to locate your manuscript file.

Please include the following items when submitting your revised manuscript:

- A rebuttal letter that responds to each point raised by the academic editor and reviewer(s). You should upload this letter as a separate file labeled 'Response to Reviewers'.
- A marked-up copy of your manuscript that highlights changes made to the original version. You should upload this as a separate file labeled 'Revised Manuscript with Track Changes'.
- An unmarked version of your revised paper without tracked changes. You should upload this as a separate file labeled 'Manuscript'.

If you would like to make changes to your financial disclosure, please include your updated statement in your cover letter. Guidelines for resubmitting your figure files are available below the reviewer comments at the end of this letter.

If applicable, we recommend that you deposit your laboratory protocols in protocols.io to enhance the reproducibility of your results. Protocols.io assigns your protocol its own identifier (DOI) so that it can be cited independently in the future. For instructions see: <http://journals.plos.org/plosone/s/submission-guidelines#loc-laboratory-protocols>

We look forward to receiving your revised manuscript.

Kind regards,

Muhammad Hussnain Siddique
Academic Editor
PLOS ONE

Journal Requirements:

When submitting your revision, we need you to address these additional requirements.

1. Please ensure that your manuscript meets PLOS ONE's style requirements, including those for file naming. The PLOS ONE style templates can be found at

https://journals.plos.org/plosone/s/file?id=wjVg/PLOSOne_formatting_sample_main_body.pdf and

<https://journals.plos.org/plosone/s/file?id=ba62/PLOSOne_formatting_sample_title_authors_affiliations.pdf>

-> Thank you for your comments. We corrected our manuscript, especially the front page, to meet the PLoS One’s style requirements. For example, we removed the street addresses from the affiliations and the physical addresses from the corresponding authorship, and added the corresponding authors’ initials.

2.  We note that the grant information you provided in the ‘Funding Information’ and ‘Financial Disclosure’ sections do not match.

When you resubmit, please ensure that you provide the correct grant numbers for the awards you received for your study in the ‘Funding Information’ section.

  Thank you for the comments. We provide the correct grant numbers.

3. PLOS ONE now requires that authors provide the original uncropped and unadjusted images underlying all blot or gel results reported in a submission’s figures or Supporting Information files. This policy and the journal’s other requirements for blot/gel reporting and figure preparation are described in detail at https://journals.plos.org/plosone/s/figures#loc-blot-and-gel-reporting-requirements and https://journals.plos.org/plosone/s/figures#loc-preparing-figures-from-image-files. When you submit your revised manuscript, please ensure that your figures adhere fully to these guidelines and provide the original underlying images for all blot or gel data reported in your submission. See the following link for instructions on providing the original image data: https://journals.plos.org/plosone/s/figures#loc-original-images-for-blots-and-gels.

In your cover letter, please note whether your blot/gel image data are in Supporting Information or posted at a public data repository, provide the repository URL if relevant, and provide specific details as to which raw blot/gel images, if any, are not available. Email us at plosone@plos.org if you have any questions.

-> Thank you for your comments. We have only one gel picture in the manuscript. We converted the original, uncropped gel image to PDF file, named it ‘S1_raw_images’, and uploaded as supporting information to follow the instruction provided by PLoS One.

4. Please include captions for your Supporting Information files at the end of your manuscript, and update any in-text citations to match accordingly. Please see our Supporting Information guidelines for more information: [http://journals.plos.org/plosone/s/supporting-information](about:blank).

-> Thank you for your comments. Now, we include the caption for our supporting information file, S1_raw_images under a newly created heading, ‘Supporting information’ as in line of 695-697 after revision in track changes.

[Note: HTML markup is below. Please do not edit.]

Reviewers' comments:

Reviewer's Responses to Questions

**Comments to the Author**

1. Is the manuscript technically sound, and do the data support the conclusions?

The manuscript must describe a technically sound piece of scientific research with data that supports the conclusions. Experiments must have been conducted rigorously, with appropriate controls, replication, and sample sizes. The conclusions must be drawn appropriately based on the data presented.

Reviewer #1: Partly

Reviewer #2: No

2. Has the statistical analysis been performed appropriately and rigorously?

Reviewer #1: Yes

Reviewer #2: Yes

3. Have the authors made all data underlying the findings in their manuscript fully available?

The [PLOS Data policy](http://www.plosone.org/static/policies.action#sharing) requires authors to make all data underlying the findings described in their manuscript fully available without restriction, with rare exception (please refer to the Data Availability Statement in the manuscript PDF file). The data should be provided as part of the manuscript or its supporting information, or deposited to a public repository. For example, in addition to summary statistics, the data points behind means, medians and variance measures should be available. If there are restrictions on publicly sharing data—e.g. participant privacy or use of data from a third party—those must be specified.

Reviewer #1: Yes

Reviewer #2: Yes

4. Is the manuscript presented in an intelligible fashion and written in standard English?

PLOS ONE does not copyedit accepted manuscripts, so the language in submitted articles must be clear, correct, and unambiguous. Any typographical or grammatical errors should be corrected at revision, so please note any specific errors here.

Reviewer #1: Yes

Reviewer #2: No

 -> We had our manuscript edited by a paid editing service. We uploaded the editing certificate.

5. Review Comments to the Author

Please use the space provided to explain your answers to the questions above. You may also include additional comments for the author, including concerns about dual publication, research ethics, or publication ethics. (Please upload your review as an attachment if it exceeds 20,000 characters)

Reviewer #1: In my opinion, the ms should be revised carefully before further consideration.
1. lines 89-93: This can be removed from M&M. It's not description of methods. More like a introduction.

-> Thank you for your comments. According to your suggestion, a brief explanation on the *B. cereus* isolates was moved to introduction (line 89-90 after revision in track change), however more detail comments on the isolates, for example the numbers and sources of the isolates remain in materials and methods.

2. lines 85: What's EPS-degrading enzymes here? The authors used DNAes, protease and cellulase?

-> Thank you for the good comments. We studied six different EPS-degrading enzymes, DNase I, proteinase K, dispase II, cellulase, amyloglucosidase, and α-amylase. We added this information in the text as in line 87-88 after revision in track change.

3. Line 88: What's the definition of biofilm forming strain here? More description is needed.

-> Thank you for your comments. The biofilm forming strain is defined now as in line 119-126 after revision in track change. Basically, we followed the previous protocol of Stepanović et al. (2000).

4. Line 108: In my opinion, it's not biofilm of strain but only colony.

-> Thank you for the good comments. As the reviewer pointed out, the overnight culture shouldn’t be a biofilm. It is just a cell suspension used to form biofilms. To prevent any confusion, the sentence is now modified (line 138-139 after revision in track change) and the preparation of cell suspension is now described under a separate section, ‘Preparation of cell suspension’ (line 134-139 after revision in track change) from the section of ‘Enzymes used in susceptibility test’.

5. More advanced technology should be applied to understand the structure of colony (biofilm?) before and after enzyme applicaitn. Not only the appearacne. For example, TEM for the inner structure; Staining for protein distribution.

-> Thank you for your comments. We used confocal laser scanning microscopy (CLSM) to image the biofilms exposed to enzymes and also to differentiate protein, carbohydrate, and cells in biofilms using fluorescent dyes. Those images are provided in Figure 3 and 8.

6. Why the extracellular DNA was secreted? Should explain this.

-> Thank you for the good comments. We tried to explain the role of extracellular DNA secreted in discussion section as in line 489-492 after revision in track change.

Reviewer #2: Line 38 "In this study, B. cereus strains from tatsoi and the ATCC 10987 reference strain" it's better to add codes of different strains used in this study like "B. cereus strains from tatsoi (BC4, BC10 and BC72)...........".

-> Thank you for your comments. We made a correction as the reviewer pointed out as in line 40 after revision in track change.

It is not clear how the isolated B. cereus strains were identified and confirmed as B. cereus. Chemical method or ribotyping? Please add results.

-> Thank you for your comments. As the reviewer pointed out, method for the identification and confirmation of *B. cereus* isolates was added in the materials and methods section of the revised manuscript. During the revision, we’ve found mistake in reference #18 (initial submission) and have changed as correct one (reference #18 after revision in track change). The added sentences on the identification and confirmation of *B. cereus* isolates were as follow (Line 99 - 110 in the revised manuscript after track change): To isolate *B. cereus* and *B. thuringiensis*, Polymerase chain reaction (PCR) amplification of the crystal (*cry*) gene was carried out for the biochemically identified *B. cereus* group isolates [Reference #19]. Biochemical identification for the B. cereus group was conducted by using the Vitek-II system with the BCL card (bioM´erieux, Inc., Marcy l’Etoile, France), according to the manufacturer’s directions. The specific primer pair K3 (5’-GCTGTGACACGAAGGATATAGCCAC-3’) and K5 (5’-AGGACCAGGATTTACAGGAGG-3’) was used for the identification of the *cry* gene (1,600 to 1,700 bp) for *B. thuringiensis*. Template DNA was preheated at 94℃ for 7 min. Then, the DNA was denatured at 94℃ for 60 s and annealed to primers at 58℃ for 90 s; and the PCR products were extended at 72℃ for 60 s for 30 cycles for the *cry* gene (ref #19, ref #20).

Line 123-124: Specify the staining solution?

-> Thank you for your comments. We removed CV which is an abbreviated form of crystal violet and mentioned the full name with more detailed description in the text to make it clear as in line 161-162 after revision in track change.

Which device was used for imaging Biofilm morphology?

-> Thank you for your comments. The images were captured by a commercial digital camera (EX2F, Samsung, Korea) and this information is added to the text as in line 217-218 after revision in track change.

Study Biofilm inhibition lacks reference/s. Authors are advised to mention reference clearly which was used for this study.

-> Thank you for your comments. We added the reference (Ref. 23) we followed to study biofilm inhibition and removal as in line 154-155 after revision in track change.

Line 197 "and 1 environmental isolate (strain BC72), strains BC4 and" replace "," with full stop and make two sentences.

-> Thank you for your comments. We made a correction according to the reviewer’s comment as in line 267 after revision in track change.

Line 377-389 "eDNA plays an important role in biofilm formation................ B. cereus strains in the environment". Figure 2 suggests that BC4 is susceptible to DNAse I, It is not clear why eDNA was not detected in biofilm of BC4? Suggest plausible reason.

-> Thank you for the good comments. Unfortunately, we do not have any clue why we are not able to detect eDNA of BC4 biofilm by running purified DNA on agarose gel. We just assume that eDNA of BC4 could be low molecular weight with a long range of size and may not be visible on the gel. However, it is still interesting that any DNA molecules not protected by impermeable membranes, which look red, seems to be degraded by DNase I as shown in Figure 3. It might suggest that eDNA could exist in BC4 biofilm.

It seems something was wrong with the data in figure 2C. The absorbance of crystal violet in the absence of degrading enzyme (-) (fig2C) is not comparable with the corresponding absorbance of crystal violet in fig 1A. Please comment.

-> Thank you for your point out. Currently, we do not understand why such a discrepancy occurred. However, it is true that experiment-to-experiment variation exists in biofilm experiments.

Line 233-234: Why some enzymes promoted biofilm formation in a strain-dependent manner (Fig 2A and 2C).

-> Thank you for your comments. We hypothesize that in some cases the strains may not have the substances targeted by the exogenous enzymes or have the targeted substances protected. In addition, they may be able to degrade them by using the self-secreted protease enzymes. The degraded exogenous enzymes could serve as a nutrient and promote the biofilm formation. Therefore, the strain-dependency in promotion of biofilm formation could be due to strain-dependent variation in both EPS composition and self-secreted enzymes (ex. *Staphylococcus aureus*) (Kiedrowski et al., 2011).

Careful English editing is advised, help from a native-language expert or paid services shall be taken.

-> Thank you for your advice. Our manuscript is edited by an English editing service. The editing certificate is uploaded.

The word "in addition" in line 74 shall be replaced by an alternative word as it was previously mentioned in line 46 and then in line 82 again.

-> Thank you for your comments. The word “in addition” in line 74 has been changed to the word “Particularly”, considering the context, as in line 76-77 after revision in track change.

Line 76: Word “although” is not required.

-> Thank you for your comments. We removed the word “although” from the sentence as the reviewer pointed out.

Line 97-99 “Among the B. cereus .......... seeds and environmental samples” can be written as “Among the B. cereus isolates, ones with highest biofilm formation abilities were selected from each category i.e. strain BC4 from microgreen samples, BC10 from seeds of microgreens and BC72 form water samples were selected for current study”.

-> Thank you for your comments. As the reviewer suggested, we rephrased the sentence as in lines 127-133 after revision with track changes.

6. PLOS authors have the option to publish the peer review history of their article ([what does this mean?](https://journals.plos.org/plosone/s/editorial-and-peer-review-process#loc-peer-review-history)). If published, this will include your full peer review and any attached files.


If you choose “no”, your identity will remain anonymous but your review may still be made public.


**Do you want your identity to be public for this peer review?** For information about this choice, including consent withdrawal, please see our [Privacy Policy](https://www.plos.org/privacy-policy).

Reviewer #1: No

Reviewer #2: **Yes:** Muhammad Khurshid

[NOTE: If reviewer comments were submitted as an attachment file, they will be attached to this email and accessible via the submission site. Please log into your account, locate the manuscript record, and check for the action link "View Attachments". If this link does not appear, there are no attachment files.]

While revising your submission, please upload your figure files to the Preflight Analysis and Conversion Engine (PACE) digital diagnostic tool, <https://pacev2.apexcovantage.com/>. PACE helps ensure that figures meet PLOS requirements. To use PACE, you must first register as a user. Registration is free. Then, login and navigate to the UPLOAD tab, where you will find detailed instructions on how to use the tool. If you encounter any issues or have any questions when using PACE, please email PLOS at [figures@plos.org](mailto:figures@plos.org). Please note that Supporting Information files do not need this step.

*In compliance with data protection regulations, you may request that we remove your personal registration details at any time.* [*(Remove my information/details)*](https://www.editorialmanager.com/pone/login.asp?a=r)*. Please contact the publication office if you have any questions.*
